# Using Core Elements of Health and Safety Management Systems to Support Worker Well-Being during Technology Integration

**DOI:** 10.3390/ijerph192113849

**Published:** 2022-10-25

**Authors:** Emily J. Haas, Emanuele Cauda

**Affiliations:** 1 National Personal Protective Technology Laboratory, National Institute for Occupational Safety and Health, Pittsburgh, PA 15236, USA; 2 Pittsburgh Mining Research Division, National Institute for Occupational Safety and Health, Pittsburgh, PA 15236, USA

**Keywords:** direct reading and sensor technologies, health and safety management systems, occupational safety and health, total worker health, well-being

## Abstract

Research studying the intersection of occupational safety and health (OSH) and direct reading and sensor technologies (DRST) is sparse, with a specific lack of research available that has empirically considered ways that DRST may impact worker well-being. In this paper, the authors examine how organizations could utilize core elements of their health and safety management system (HSMS) to coordinate and execute DRST in the workplace to support worker well-being. National Institute for Occupational Safety and Health (NIOSH) researchers developed a 39-item questionnaire targeting OSH professionals to understand attitudes toward DRST and the current and intended uses of DRST at their place of employment. Eighty-eight OSH professionals completed the questionnaire between August and December 2021. Descriptive results of the study sample are provided but the focus of the study applies the open-ended responses to two questions, which was deductively analyzed. Descriptive results show that reliability and validity of data was a top concern while the open-ended qualitative feedback revealed three primary themes: (1) acceptability and trust in technology; (2) ease of use; and (3) support and guidelines. Results provide an opening to use core HSMS elements (i.e., management commitment and leadership, communication and coordination, and employee involvement) during DRST integration to demonstrate support for workers during times of ambiguity and change.

## 1. Introduction

Research studying the intersection of occupational safety and health (OSH) and smart technologies is sparse [1]. Although OSH is seldomly included as a primary goal of Industry 4.0 (also termed the Fourth Industrial Revolution) [2], many of these technologies inherently manage worker safety and health (S&H) in hazardous workplaces [3,4]. For example, the use of direct reading and sensor technologies can detect and measure worker exposure to contaminants including gas, vapor, aerosols, and fine particulates, and can monitor physical hazards respective to workers and equipment [5]. Further, direct reading and sensor technologies have demonstrated utility in detecting and monitoring hazardous conditions or individual factors that may pose risks to workers such as fatigue monitoring and detection and identifying potential interventions to mitigate these S&H risks [6].

Previous research has identified concerns over cost, confidentiality, and information overload when integrating new technologies in the workplace [7]; however, little information is available regarding the perceived barriers that organizations and their respective OSH professionals may have on their cohesive, widespread implementation [8]. There is a lack of research available that has empirically considered ways that smart or advanced technologies may impact worker well-being and mental health [9]. However, research articles and literature reviews have highlighted the potential, negative impacts of these technologies on worker mental health and well-being [10].

For example, Johnson and colleagues [10,11], argue that these technologies are designed to increase productivity and organizational metrics without considering employee outcomes. Studies have shown that the introduction and presence of such technologies can create or foster workplace norms that are linked to unrealistic performance expectation and increased mental overload [12]. Along these lines, a plethora of studies have documented that any new technology can amplify work pace and is associated with increasing worker stress, overload, and burnout [13,14,15]. Finally, research has shown that the introduction of advanced technologies, including those that use forms of artificial intelligence, are often perceived as a threat to employees’ jobs and can negatively impact well-being [16]. Consequently, an empirical examination of employee perceptions and implementation practices is necessary to support organizational adoption and employee confidence in new technologies [17] and to preserve the well-being of workers [18].

In addition to the absence of worker well-being and new technologies being linked in the literature, the application of health and safety management systems (HSMS) to integrate and implement new technologies is also vague [1,19]. In this paper, the authors propose that if organizations use their existing HSMS to coordinate and execute guidance that supports worker engagement and consistent communication about new technologies, there may be fewer opportunities for misinformation. This topic is explored in the current paper with the overarching objective of supporting worker well-being as the future of work via new technologies continues to evolve.

Even though a variety of specific technologies and processes have been developed and are associated with advancements made with respect to Industry 4.0, for the purposes of this study, we use the term direct reading and sensor technologies (DRST) throughout the paper. This acronym is being used to reference all instruments that can monitor and alert workers’ exposure to contaminant health hazards and physical safety hazards, including individual risks on the job. Examples of technologies that encompass this broad term include direct-reading methodologies or instruments such as field-portable gas chromatographers for the analysis of gas canisters; noise dosimeters to measure personal noise exposure levels; real-time monitors such as an aerosol device that uses counting particles to continuously measure the mass concentration of a contaminant; and wearable monitors that may collect physiological or biometric data points. Fatigue monitoring and detection technologies also fall within DRST and can be used to either predict fatigue based on workers’ previous sleep and work hours or can be used to monitor and detect current fatigue by using biological and performance measures [20]. Based on the monitoring and feedback provided, DRST can be used to indicate when personal protective equipment (PPE) needs to be donned or can be safely donned.

This paper begins with an overview of HSMS elements across regulatory and non-regulatory programs. Then, the role of HSMS in introducing and integrating DRST is presented as well as possible implications on worker well-being by using such a systems approach. Then, we introduce a 2021 questionnaire of industrial hygienists and other OSH professionals with experience using DRST in the workplace. Eighty-eight individuals voluntarily completed the questionnaire, and these results were used to compare individual experiences with DRST and the perceived experiences of the organization where they were employed. Both descriptive and open-ended data are presented in a way that elucidate key barriers to implementing DRST in the workplace and what role an HSMS can have in alleviating some of these barriers. Based on the differences in perceptions between these two groups, we argue that an integrated systems approach is needed when introducing new technology to support worker well-being.

### 1.1. Occupational Health and Safety Management Systems

An HSMS is defined as a set of institutionalized, interrelated, and interacting elements strategically designed to establish and achieve OSH goals and objectives [21]. Thorough and visible HSMS programs have been linked to a reduction in injuries within many high-hazard industries, e.g., [22,23,24]. A regulatory standard for an HSMS does not exist in the U.S., but several exist internationally. In 2013, the International Organization for Standardization (ISO) pursued a global standard [25] for occupational HSMS to protect employees [26]. According to the ISO 45001 [25] standards, the purpose of an HSMS is to “provide a framework for managing health and safety risks and opportunities” and provide a “strategic and operational decision for an organization” (p. vi). It is worth noting that ISO’s updated 2018 standard added content around management commitment and leadership, workplace participation and engagement, integrated risk-based management, and culture [27,28].

**Figure 1:**
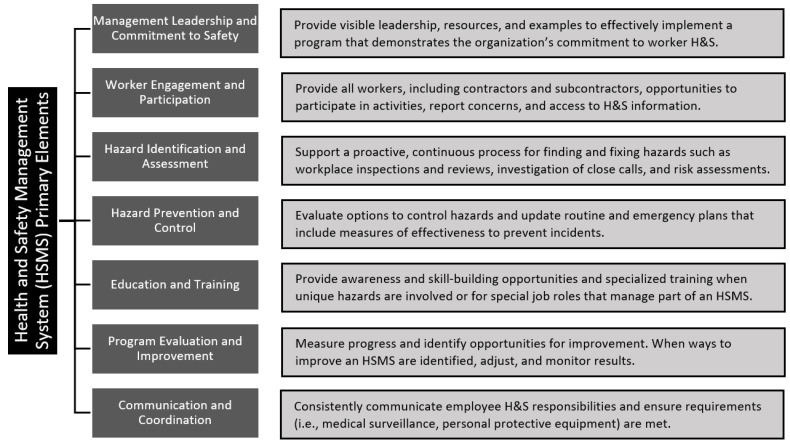
OSHA’s health and safety management system elements and example best practices (adapted from OSHA [33]).

Several HSMS frameworks are available for companies to consult and tailor as needed. These frameworks are based on a continuous improvement process to control risks to an acceptable level [29] and advocate some form of the Plan-Do-Study/Check-Act process [30,31,32]. The Occupational Safety and Health Administration (OSHA) published guidelines for recommended practices for safety and health programs that contain seven interrelated elements [33]. These seven elements are consistent with those discussed in ISO 45001 [25] and ANSI/AIHA Z10 [21] programs and consequently are applied as a reference framework within this paper. See Figure 1 for a brief description of these elements.

Several of these elements have received additional emphasis in updated ISO guidelines. Specifically, ISO 45001 [25] states that management leadership and employee participation are fundamentally connected activities that weave through the other system elements. Additionally, successful health and safety management is reliant on effective communication up, down, and across organizations [34]. Importantly, standards organizations indicate that possessing a certified HSMS with strong leadership and employee engagement shows an organization’s commitment to ensuring good working conditions, worker health, well-being, and equity practices on the job [25,35]. Given their increasingly important focus, these elements and related best practices are discussed in more detail below and are referenced throughout this paper as core elements of an HSMS.

#### 1.1.1. Management Leadership and Commitment to Safety

Management leadership has been addressed in HSMS guidance published by ISO [25], AIHA/ANSI [21] and OSHA [33], identifying involvement and leadership from all levels of management as a core component of effective safety management. Research has shown that management leadership positively affects worker behavior whereas a lack of such commitment can increase the likelihood of accidents [36,37,38]. Meta-analyses, such as one done by Dunlap [39], identified several themes that are central to strong leadership. One theme was accountability which includes managers taking responsibility for safety [40] and holding others accountable for safety [41]. Dunlap [39] also identified the need for management engagement in safety activities.

#### 1.1.2. Worker Engagement and Participation

Engagement is active participation or a high degree of involvement from employees in health and safety-related activities. ISO 45001 [25] notes involvement in decision making as being critically important, whereas ANSI Z10 highlights the importance of employees being involved in safety committees and offering ideas to improve health and safety. Studies have shown that employee engagement levels are linked to employee on-the-job injury status [42,43]. According to an empirical analysis of safety management practices completed by Dodge Data and Analytics [44], worker involvement was the most widely recognized component of a top-tiered safety system, selected by 85% of surveyed employees. It is the responsibility of senior-level leadership and frontline managers to actively work together to reduce barriers to worker participation. OSHA [33] provides examples to encourage worker involvement such as participation in programs, reporting S&H concerns, being given access to S&H information, and removing barriers to participation.

### 1.2. Considering Core HSMS Elements When Integrating New Technologies

HSMS can effectively manage health, safety, and risks in the workplace and are used by organizations to drive continuous improvement [24]. Taking this systems approach into consideration is essential when introducing new programs, tools, or practices. Research has indicated that the use of HSMS components when integrating DRST can be useful to help monitor safety performance and metrics [19]. However, scholars have argued that traditional HSMS are inflexible, and that research needs to improve the agility of current management systems to accommodate new technologies [1]. Consequently, little information is available about the ways a continual improvement process, as boasted within an HSMS, may inform effective integration of DRST in the workplace. To summarize, research has failed to formally connect HSMS and DRST in a way that supports worker health and safety [19]. This failure to update and consult core HSMS elements, principles, and complementary practices may be hindering employee support for these new technologies. Additionally, the inflexibility of some HSMSs could be at odds with the novelty and perhaps the volatility of DRST markets.

### 1.3. Potential Implications of a Systematic Integration on Worker Well-Being

Well-being is inclusive of different kinds of health such as physical, mental, emotional, and social [45,46,47]. Integrated health and safety, or well-being, is the combination or area of overlap in health protection and health promotion [46,47]. To date, worker well-being has been linked to smaller parts of the organization (i.e., a unit, crew, or discipline) which tends to produce isolated strategies, tools, and interventions for workers such as user training for new technologies. Specifically, the National Safety Council (NSC) [46] argues that well-being efforts are often introduced in piecemeal to employees and rarely integrated into the HSMS. Other research has shown that many new technologies and programs are implemented at the department level of workplaces with no guidance about the consistent implementation of programs across the workplace [48]. A problem with these disparate approaches is that they are not strategic, measurable, or connected to the HSMS whereas comprehensive HSMS practices have also been shown to support worker health and well-being [49]. This proposed, integrative approach has not been adequately considered when introducing DRST despite arguments for the development of such a safety, health, and well-being management system to identify and mitigate employee concerns [46,50]. Identifying ways to systematically incorporate DRST is incredibly important to maintaining worker well-being, especially considering findings from previous research, which has shown that the introduction of new technologies without end users in mind can cause uncertainty and lack of trust that negatively impacts not only adoption but also safety behaviors [51,52].

### 1.4. Research Questions

A more inclusive approach to integrating DRST at all levels of an organization, as linked by the HSMS, may help advance worker well-being and even productivity [53,54]. This is an important missing link and connection to explore prior to widespread DRST implementation. Data collected from industrial hygienists and OSH professionals in 2021 is used to explore this topic in more detail with the goal of alleviating worker stress and promoting well-being during the integration of new DRST. Researchers compared individual and organizational concerns around integrating OSH-specific DRST in the workplace. Specifically, the questions of interest were:

Research Question 1: Using descriptive questionnaire data, what are the differences between individual respondent concerns and perceived organizational concerns around adopting DRST in the workplace?
Research Question 2: Using open-ended feedback, in what ways can core HSMS elements (i.e., management leadership and worker engagement) be leveraged to support worker well-being when adopting DRST?

## 2. Materials and Methods

The NIOSH Center for Direct Reading and Sensor Technologies (NCDRST) was established in 2014 to coordinate research and to develop recommendations on the use of 21st century OSH technologies [55]. The Center recently engaged in formative research via an online questionnaire with close and open-ended questions to understand how various DRST have been used in the workplace as well as current strategies, benefits, and barriers to adoption among organizations and their employees.

### 2.1. Questionnaire

A 39-item questionnaire was developed by NIOSH researchers from the NCDRST to understand attitudes toward DRST, the current and intended uses of DRST at their place of employment, and barriers to DRST adoption. Questions were developed based on previous reports that have identified concerns around integrating DRST in the workplace [56] and the author’s personal research experience studying the implementation of DRST in the field with workers and managers [51,52,57,58]. Definitions of the four tier-based competencies and experience levels from the American Industrial Hygiene Association (AIHA) [56] were provided at the beginning of the questionnaire. Novel questions were close- and open-ended to probe concerns and advantages of DRST at both the individual user and organizational level as well as specific DRST activities and methods of interest. Many questions featured a “check all that apply” option. The purpose of the close-ended survey questions was to understand the current uses of DRST among various industries and workplaces rather than to measure specific constructs. So, internal validity of constructs was not necessary for these description items. Other items in the questionnaire were open-ended for respondents to provide qualitative feedback and are the primary units of analysis in the current study. The current paper uses results from the questions around perceived concerns to implementing DRST rather than specific uses and case examples of DRST already in practice. Table 1 shows the questions used to answer the research questions presented in this paper.

### 2.2. Recruitment and Participants

After receiving a NIOSH human subjects Institutional Review Board exemption, the questionnaire was hosted on the Centers for Disease Control and Prevention’s REDCap (Research Electronic Data Capture) platform [59]. The public link remained open 4 August to 31 December 2021. Initially, a convenience method was used to recruit respondents [60]. When presentations and webinars provided by NIOSH researchers around this topic were presented, a slide promoting the questionnaire was inserted at the end of each presentation. This was the only recruitment method used. To be eligible to participate, individual employees had to have some experience (any length) working as an OSH professional for any industry. The link was opened 127 times. Of those 127 instances, 39 did not proceed to answer the questionnaire while 88 (69.3%) consented and completed at least half of the questionnaire.

### 2.3. Analysis

Data were exported from CDC’s REDCap project portal into SPSS 26 [61] to clean missing data, check for outliers, and recode or collapse variables as necessary. The data file did not contain any identifying information of the individual or their place of employment. Analysis occurred in two phases. First, descriptive statistics were performed using SPSS 26 [61] to characterize the frequency and percentage of responses from those who participated.

Second, open-ended data was inductively analyzed [62]. The open-ended feedback was uploaded into Nvivo V12 [63] to aid with identification, organization, and saturation of the main themes. An inductive approach, without a framework or theory in mind, was important to embrace considering that the perceptions around DRST in the workplace have not been regularly studied. An inductive, thematic analysis of qualitative information is a method [64,65] “for identifying, analysing, and reporting patterns (themes) within data” [66], (p. 79). This approach is particularly useful when making comparisons across different groups’ experiences [66,67,68].

Upon the lead author identifying saturated themes [63,64,65,66,67,68], these were shared with the second author for review and feedback. No discrepancies arose between the two authors, but conversations were able to elucidate the overarching themes in this feedback and make connections between the feedback that was relayed on behalf of the individual and the feedback they were projecting by their organization’s stance on DRST.

## 3. Results

### 3.1. Descriptive Statistics

As previously indicated, approximately 70% of those who clicked on the public link consented and completed the questionnaire (n = 88). Of the respondents, 63.6% were male, 27.3% were female, and 9.1% preferred not to answer. Of those that responded, 29.4% were 29–39 years old; 20.0% were 40–49; 23.5% were 50–59; and 27.1% were over 60 years old. Regarding education, 5.7% possessed an associate degree or trade certificate, 25% a four-year college degree, 67% a graduate or other advanced degree, and 2.3% preferred not to answer. The respondents were primarily experts in their field with ample experience in OSH industrial hygiene and using DRST. A variety of industry sectors were represented, although only those industry sectors that had eight or more responses are reported separately. Specifically, manufacturing, mining, and oil and gas and petroleum are shown separately in Table 2 whereas the remaining industry sectors had seven or fewer responses and were added into one category. This was done to comply with IRB requirements. See Table 2 for a breakdown of the sample in these areas.

Table 3 shows the percentage of respondents who noted a specific concern related to DRST in the workplace. Reliability and validity of data received was the top concern noted by individual respondents whereas they felt the organization’s top concern was the additional cost investment of DRST in the workplace. Standardization of data was a high concern noted or perceived in both groups while individual respondents perceived issues around user acceptability, ease of use, and integration with other non-DRST technologies to be barriers to widespread implementation and use. These results are debriefed and further contextualized in the Discussion section.

### 3.2. Qualitative Themes in Respondent Feedback

Previous research has noted prevalent barriers to integrating DRST in the workplace, often referencing fears over increasing costs to purchase technologies and then retrain employees on its use to effectively implement it [69]. Although cost of investment was checked as a concern for widespread implementation in the questionnaire results, this topic was not prevalent in the open-ended feedback from respondents. Rather, the qualitative responses revealed several themes that are not well documented in the literature and can be used to glean potential barriers to future DRST use regarding impacts on worker well-being. Results also provide an opening to use HSMS practices to advance integration initiatives with worker well-being in mind. These themes include (1) acceptability and trust in the technology; (2) ease of use; and (3) support and guidelines. Each theme is briefly discussed and summarized in Table 4.

#### 3.2.1. Acceptability and Trust in DRST

Open-ended feedback commonly referenced concerns around trust in DRST from several angles. First, a lack of trust in the data produced by these technologies was a trend in feedback that mirrors the descriptive responses (Table 3), where 58% of respondents noted this was a personal concern for them in the workplace. Among individual feedback, respondents noted concerns around data privacy being secured as well as ensuring the data was valid and reliable. Second, another root cause affecting DRST trust and acceptability was employees not understanding or believing the purpose of using DRST in the workplace. Respondents noted that employees think these technologies are listening or recording their productivity or what they say on the job, causing stress and anxiety. From an organizational perspective, respondents felt that their employers did not fully understand the S&H benefits of DRST. This lack of awareness or buy-in of the benefits was perceived as a primary inhibitor for their employers’ purchasing and using DRST. Examples of feedback provided are listed in Table 4.

#### 3.2.2. Ease of DRST Use and Integration

Another theme that developed among the feedback was the overarching challenge of using either one or many technologies in the workplace. Examples included training and retraining employees on not only how to use DRST but also how to interpret alarms or alerts, and then how to respond to the alarms or alerts that are triggered. For example, one respondent noted, “Persuading management and staff to learn how to use instruments is often a big roadblock.” Other respondents took this concept further in noting the challenge of getting non-IH employees to not only use and understand these technologies but to do so with little oversight and feedback, as most IH positions oversee several work operations and cannot always be available for support. 

From an organizational perspective, respondents felt that their employers can be easily disappointed because not all DRST processes and uses are the same. As one individual noted, “Those [organizations] who see a benefit generally draw parallels to gas monitoring and are disappointed when a deployment is not as simple. Benefits are not realized, and the program is shelved.” Along these lines, individuals often felt that integrating DRST and making sense of the data to communicate implications to upper management was a challenge.

#### 3.2.3. DRST Support, Guidelines, and Best Practices

Last, there was an abundance of feedback from respondents that focused on the lack of DRST guidelines available that could provide support for using technologies in the workplace as well as guidelines or best practices for their use. For example, one respondent noted that “getting regulatory bodies to accept data” was the biggest barrier to DRST adoption. Along a similar vein, one respondent noted that, without external support from governing bodies to use DRST for certain monitoring, that DRST just becomes one more thing that people must do. For example, one respondent noted, “Some of the testing we do using non-DRST is required by law. DRST might give more time-sensitive data, but in some cases, we are required to back up our analysis by more traditional testing means.” Consequently, several respondents noted the additional time and steps it takes to work through the applications they must use and the applications they want to use.

## 4. Discussion

**Figure 2:**
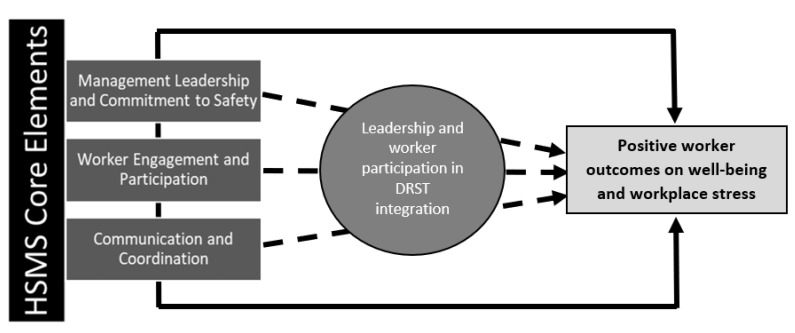
Conceptual model showing the application and impact of core HSMS elements during DRST integration to support worker well-being.

This study reported on the feedback provided by 88 industrial hygienists and other OSH practitioners via an anonymous questionnaire. Responses reiterated and revealed concerns about widespread DRST applications in the workplace, many of which focused on the prospective or actual impact and trepidation among the workforces. Although trends in the quantitative feedback were more divergent between the perspectives of individuals and those projected to the organizational level, the qualitative responses shared consistent barriers between the two. Feedback is further summarized to encourage thinking around how these perceptions and experiences may impact worker well-being, and to interpret the possible role of HSMS core elements in mitigating negative outcomes during new technology integration. Figure 2 conceptually shows the interpretation of results that are further contextualized in the discussion before showing what organizations can do with these results to support worker well-being during DRST integration.

### 4.1. HSMS Contributions to Support Worker Well-Being When Introducing DRST

Notably, these results show that individual employees have more concerns about the future use of DRST in their workplace than what they perceive their employers to have. This perceptual difference illustrates a potential disconnect between organizational processes for implementing DRST and the employees who oversee or work with those who use this technology. Specifically, open-ended responses pointed to perceived or experienced stress and anxiety among end users when first using DRST. The results indicate that organizations should be transparent about their reasons for introducing any new DRST. The NSC [46] notes that whenever anything new is implemented, showing genuine care about the well-being of workers is important. Specifically, practitioners have argued that, if organizations can execute the S&H practices outlined in standards such as ISO 45001, it can send a powerful message to employees that the organization cares for its workers [25].

When organizations integrate DRST they are likely to consult and use technical practices outlined in their HSMS such as engineering controls and auditing to ensure minimum regulatory criteria are met [70], but they may less often consider those core elements discussed throughout this paper. This oversight is notable in the results herein with a lack of trust, management support, and inconsistent guidelines and communication emerging as common barriers to using DRST. To this end, the results provide an impetus for organizations to preemptively develop site-specific guidelines and best practices to plan for, test, and evaluate DRST in the workplace. Guidelines and best practices may communicate the advantages and purpose of DRST and involve employees in what their data means in relation to personal S&H.

Research has argued that the future adoption of technologies is dependent on the ability of OSH professionals to address identified barriers to acceptance [8,17]. Fortunately, results of this study show that several identified barriers are within the control of organizations, who can use their HSMS to help structure and control the consistent narrative and implementation of technologies to not only promote adoption but also support worker well-being. Specifically, the use of the core HSMS elements highlighted in the Introduction (i.e., management commitment and leadership, communication and coordination, and employee involvement) can play a critical role in demonstrating support for workers during these times of change and learning.

#### 4.1.1. Management Commitment and Leadership

The descriptive results showed that data reliability and validity are of greater concern among employees, likely hindering trust and acceptability of DRST. The qualitative feedback also indicated that some employees may question the true intention of DRST in the workplace. Previous research supports this result, with one study showing that worker perception of being monitored is the biggest worry in using advanced technologies in the workplace [17]. If the introduction and explanation of DRST is made without the worker in mind, employee doubts and anxiety may increase. For example, Heger and colleagues [71] argued that trust-based user acceptance is particularly important for the more intrusive applications such as affective computing.

Based on the current study results, managers’ commitment to integrating technologies is increasingly important for employees to visibly see to increase their trust in new DRST. Specifically, if management does not offer a plan that commits to the purpose of new technologies, then the use of technologies tends to decrease after implementation, thereby negatively impacting worker perceptions of OSH [72]. It has been suggested that whenever health information is collected in the form of data, the data should be analyzed in aggregate form only and shared back with employees [46]. By minimizing individual-level data that is seen by the organization, workers may be more receptive to use such technologies. Further, when the aggregate form only approach is used, managers can share results and fix problems more quickly [73], which can build and foster trust among the workforces.

#### 4.1.2. Worker Engagement and Communication

Worker engagement is linked to many positive outcomes, including a reduction in injuries experienced on the job [43]. Alternatively, this same study showed that those with no job-related injuries reported higher levels of engagement and communication with their supervisors. Consequently, within the planning phase of any new HSMS program or practice, it is important to create opportunities for employee involvement to give workers more control and autonomy in the workplace [2]. This involvement process includes clear communication and guidance to workers who may be affected by the integration of new DRST. Additionally, flexible communication while DRST is in use by workers is also important to ensure worker safety and health [73]. Such involvement can help prevent potential incidents around DRST in the workplace and increase worker trust in DRST. Finally, involving workers in feedback around DRST may inform and improve the design of these products in the future.

Previous research has shown that employees welcome employer engagement in their personal health, particularly guidance and support in workplace health promotion initiatives [74]. Consequently, it is important for organizations and management to involve employees in the development and implementation of new policies and procedures when introducing DRST. Specifically, positive messages were provided by respondents regarding the necessary role and positive outcome of employee involvement. One respondent stated, “Employees, in general, can be great participants in the exposure monitoring effort if they are approached in an honest, responsible manner AND are provided with the results at the end of the monitoring period.” Involvement in these processes could improve employee buy-in of new technologies and help employees understand why certain procedures are important to follow.

Additionally, involving employees in this process may create a non-threatening environment where employees feel comfortable approaching management to discuss safety-related concerns. Previous research has shown that communication around the pros and cons of new technologies are necessary to support realistic expectations [2], stating, “humans need to feel that the value they get from the system is worth their effort and cooperation to make the system work” (p. 684). Other research has supported this concept, illustrating that showing workers their respirable dust exposure specifically after 2–4 h of monitoring provided them with autonomy and knowledge to make changes on the job [57]. Such transparent communication that includes a feedback loop to involve employees in their own exposure data may alleviate concerns and even empower future use of DRST.

Finally, considering how to best utilize worker engagement within an HSMS extends beyond only involving end users in this process. Organizations should also consider how to holistically expand and involve relevant departments throughout the DRST integration processes. For example, participants noted that individuals in IT to help download the data or information from manufacturers about how to interpret the data, may be useful. Such feedback illustrates that the involving workers in feedback mechanisms may not only improve user experiences and adoption but perhaps inform product design as well.

#### 4.1.3. Summary

**Figure 3:**
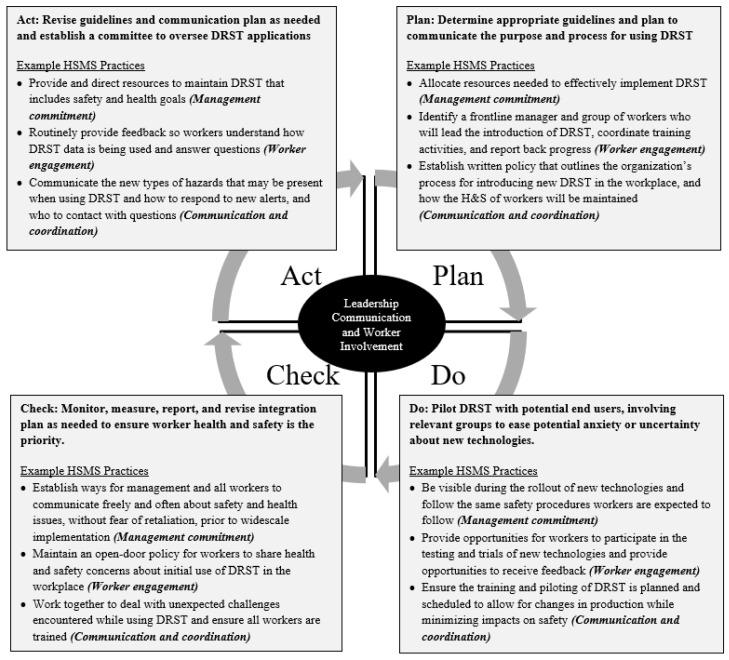
Integrated health and safety strategies via the P-D-C-A cycle within an HSMS to enhance overall worker well-being during new technology integration (example practices adapted from [33,80]).

Researchers used both the descriptive questionnaire results and open-ended feedback to identify differences in the concerns of employees and organizations regarding the use and integration of DRST. Specifically, the purpose of open-ended data is to help understand the “why” of quantitative data [75]. Although the only close-ended data used were descriptive in nature, the themes that emerged during the qualitative analysis of open-ended responses show overlap in the main areas of concern (i.e., breadth of topics), and demonstrate the depth of these concerns between individuals who oversee or use DRST and their employers. Some of these concerns or potential risks may be more effectively reduced by addressing elements within a system rather than individual behavior [76]. Specifically, current results show that an integrative approach that considers employee health protection and promotion may be more effective in establishing and sustaining the use of new technologies and the processes associated with their use [45,46,47,76,77,78]. Consequently, organizational awareness of core HSMS elements—management commitment, communication and coordination, and involvement—are important to consider moving forward. Using results of the current study and recommended S&H practices that have been provided by OSHA [33], example practices are provided in Figure 3 that were developed with consideration of worker well-being during new technology integration. Organizations may consider similar practices as they go through the HSMS Plan-Do-Check-Act (P-D-C-A) cycle when integrating DRST (See Figure 3). It is worth noting that the link between an HSMS and physical safety and mental health outcomes has already been made in previous research [77,78,79]. This study shows that the same application of an HSMS is important when integrating new DRST to also support worker well-being on the job. Figure 3 shows the application of such validated HSMS practices [80] when considering this integration process.

### 4.2. Limitations

Although the suggested focus on core HSMS elements and respective practices lays the foundation for a relevant roadmap to consider when integrating new technologies with worker well-being in mind, this does not come without limitations. First, this was a convenience sample of respondents who were aware of the public questionnaire due to their attendance at one or more virtual conferences in 2021. We did not aim to recruit a representative sample across industry or experience levels. However, one advantage of this convenience sample is that most respondents reported being highly skilled in DRST, increasing the likelihood of relevant barriers that may be encountered during the integration process.

Additionally, the feedback, although anonymous, is still subject to social desirability bias regarding the individual feedback and the projected organizational barriers that were shared. Because the adoption among professionals in industrial hygiene and OSH has generally been complicated (e.g., related to the accuracy of the field-based methods, the complexity in the adoption, and the cost) [5], additional data that is more representative of industry experience on behalf of individual users (i.e., workers) and the organization is still needed.

More specifically, because this study primarily relied on qualitative feedback to explore potential links between HSMS, DRST, and implications for worker wellbeing, it is important for quantitative data to explore this issue more explicitly in the future. For example, we did not inquire about the presence or absence of an organization’s HSMS or how it was used, if at all, during the integration of new DRST. Additionally, because this data is a snapshot in time, we cannot state that our proposed results would indeed impact worker wellbeing. For these reasons, it is unclear whether organizations did or did not consider their HSMS as a tool to ensure worker wellbeing when implementing DRST. However, because this study was focused on ensuring HSMS elements and practices were used when integrating new technologies, these limitations do not impact the utility of the current study.

## 5. Conclusions

Although the primary objective of the questionnaire was to assess the current perceptions and use of DRST among professionals, the results were insightful in providing strategic guidance to the Center and considerations for mindful DRST integration. A variety of DRST applications have become important tools for industrial hygienists and OSH professionals [81,82]. To date, more research has been conducted around perceptions of DRST pre-introduction and not necessarily during or post-integration to better understand the paradoxes of these devices in OSH. Uniquely contributing to the literature, this study revealed possible root cause challenges during DRST implementation that negatively impact employee perceptions and adoption. Although smart technologies associated with Industry 4.0, such as DRST, can improve work processes and protect aspects of the work environment, the adoption of these technologies in high-risk industries is still in its early stages [83]. This early stage of integration and adoption means that there is still time to systematically consider best methods for DRST integration. Specifically, worker well-being as a documented focus when selecting, implementing, and evaluating DRST applications is essential.

When examining the integration of technology, people, and processes, some have argued that S&H is still not well represented [35]. Additionally, Lee and Johnstone [2] argued that, to date, new technologies within Industry 4.0 have been developed in isolation without consideration of the organizational culture that is part of an HSMS. However, the integrated use of an HSMS has not been holistically studied [19]. The implications from this study show that the inclusion and use of an organization’s core HSMS elements may be the missing link to continually ensure that S&H is considered when integrating new technologies in the workplace. Moving forward, results show the need for updated and flexible policies and practices, which can be facilitated through core elements of an HSMS, to support employee well-being as the future of work continues to evolve.

## Figures and Tables

**Table 1:** Descriptive and open-ended concerns toward DRST adoption in the workplace.

What concerns ***do you have*** about adopting DRST in the workplace (check all that apply)?	What concerns does ***your organization*** have about adopting DRST in the workplace (check all that apply)?
Reliability and validity of data received Standardization of data Ethical considerations Legal considerations User acceptability Integration of data with non-DRST methodologies Complexity of use Lack of time to learn Additional cost of investment IT governance Please expand on concerns with specific examples_____________________________________	Reliability and validity of data received Standardization of data Ethical considerations Legal considerations User acceptability Integration of data with non-DRST methodologies Complexity of useLack of time to learn Lack of time to learn Additional cost of investment IT governance Please expand on concerns with specific examples_____________________________________
Describe ***personal concerns*** you have with using DRST in the workplace. (open-ended)	Describe the ***concerns of your organization*** with using DRST in the workplace. (open-ended)

**Table 2:** Respondent sample industrial hygiene, industry, and DRST experience.

Categorical Experience-Based Variables	N	Percentage
**Experience in OSH Industrial Hygiene**	N = 85	
Less than 10 years	14	16.5%
11–15 years	17	20.0%
16–20 years	12	14.1%
More than 21 years	42	49.4%
**DRST Tier (AIHA definition)**	N = 60	
Tier 1 (Basic) and Tier 2 (Intermediate)	7	11.7%
Tier 3 (Specialist)	23	38.3%
Tier 4 (Advanced)	30	50.0%
**Industry**	N = 86	
Manufacturing	11	12.8%
Mining	9	10.5%
Oil and Gas, Petroleum	8	9.3%
Multiple and or Other Sectors (e.g., Transportation, Agriculture, Construction, Healthcare and Social Assistance)	58	67.5%

**Table 3:** Concerns about using DRST in the workplace.

DRST Concern	Respondents’ DRST Concerns (%)	Perceived DRST Concerns of Respondents’ Employers (%)
Reliability and validity of data received	58.0	33.3
Standardization of data	31.8	20.5
Ethical considerations	8.0	3.4
Legal considerations	17.0	17.0
User acceptability	30.7	14.8
Integration of data with non-DRST methodologies	30.7	15.9
Complexity of use	30.7	20.5
Lack of time to learn	10.2	13.6
Additional cost of investment	29.5	39.8
IT governance	14.8	14.8
Do not understand the benefits provided by DRST	1.1	6.8

**Table 4:** Themes in concerns expressed by respondents toward using DRST in the workplace.

Personal Concerns and Barriers Experienced with DRST in the Workplace	Perceived Organizational Concerns and Barriers Experienced with DRST in the Workplace
***Theme 1: Trust and acceptability***	
Need to ensure data privacy is upheld and ensure this is considered when using this technology with workers, especially when coupled with video exposure monitoring User acceptability, and reliability + validity of data. Employees believed we were recording or listening to what they were saying not just measuring noise.	Benefits are not well known by risk owners, increasing hesitation toward acceptance. Organization does not really understand the use of DRI and the role it plays in protecting workers. Convincing management and workers that monitor alarms are both a safety and health hazard.
***Theme 2: Ease of use and subsequent integration***	
The variety of instruments needed for all types of exposures creates problems for workers trying to remember which buttons to push. Properly using and managing equipment and responding to alarm conditions. Getting non-IH employees to understand how to use them with little oversight from me. It can be difficult using dosimeters because employees remove them, replace them incorrectly, cover them up, etc.	The instruments are too difficult to use and too expensive, and the results too easily misinterpreted. Persuading management and staff to learn how to use instruments. New instruments take time to learn how to use effectively. It can be hard to integrate DRI and non-DRI data in way that makes sense for upper management.
***Theme 3: Guidance—or lack thereof—to support adoption***	
Lack of regulations and consensus standards on how to operate, use, maintain DRIs. Will these methods be accepted by local OHS regulators? Misinterpretation of the data by external stakeholders (i.e., trades unions/regulators).	Without guidelines, external affairs and legal experts have raised concerns about data privacy. No regulatory support so the company looks at the cheapest way to compliance.

## Data Availability

Restrictions apply to the availability of these data. Please contact the authors for more information.
